# Personality-Activity Alignment: Implications for Dementia Risk Reduction

**DOI:** 10.1093/geroni/igaf122.127

**Published:** 2025-12-31

**Authors:** Joseph Svec, Yulri Kim, Jeong Eun Lee, Natasha Nemmers

**Affiliations:** St. Joseph’s University, Patchogue, New York, United States; Iowa State University, Ames, Iowa, United States; Iowa State University, Ames, Iowa, United States; Wayne State University, Detroit, Michigan, United States

## Abstract

Certain personality types (i.e. neuroticism) are often accompanied by higher risks of cognitive decline, whereas others of the “big five” (i.e. conscientiousness) correspond with lower risks. However, such associations largely focus on independent associations of personality types and cognitive health. We extend this literature by focusing on the connections between personality types and personal behaviors, specifically the extent to which older adults engage in social and non-social activities. Utilizing a person-environmental fit perspective, this study examines not only the associations between activity associations and cognitive decline but also whether the configurations of personality-activity dimensions amplify such activity effects. Using the Health and Retirement Study (N = 3,380), we run a series of logistic regressions results predicting dementia. Preliminary findings suggest that social activities significantly correspond with reduced odds of dementia. However, the positive and significant interaction of neuroticism and social activities (OR = 1.63) indicate that social activities have increasingly reduced benefits for those with higher levels of neuroticism. Conversely, those with the lowest conscientiousness scores, social activities increase dementia risk by 461% (OR = 5.61) with the caveat that each additional unit increase in conscientiousness scores reduce the odds of social activities by 42% (OR = 0.58). In other words, conscientiousness appears to enhance the protective effects of social activities against dementia risk. The observed relationships highlight that social engagement or activities and their alignments with personality types may represent modifiable factors associated with dementia risk. Thus, future research may benefit from more nuanced understandings of activities as conditional to personal and environmental fits.

